# A cross-sectional study of foot-ground clearance in healthy community dwelling Japanese cohorts aged 50, 60 and 70 years

**DOI:** 10.1186/s12877-021-02117-w

**Published:** 2021-03-06

**Authors:** Hanatsu Nagano, W. A. Sparrow, Katsuyoshi Mizukami, Eri Sarashina, Rezaul Begg

**Affiliations:** 1 Institute for Health and Sport (IHeS), Victoria University, P.O. Box 14428, VIC 8001 Melbourne, Australia; 2 Graduate School of Comprehensive Human Sciences, Faculty of Health and Sport Sciences, University of Tsukuba, Ibaraki 305-8574 Tsukuba, Japan

**Keywords:** Tripping, Gait, Falls, Minimum foot clearance, Ageing, Community health

## Abstract

**Background:**

Falls-related injuries are particularly serious for older people, causing pain, reduced community engagement and associated medical costs. Tripping is the leading cause of falls and the current study examined whether minimum ground clearance (MFC) of the swing foot, indicating high tripping risk, would be differentiated across cohorts of healthy 50-, 60- and 70-years old community residents in Japan.

**Methods:**

A cross-sectional population comprising the three groups (50s, 60s and 70s) of 123 Konosu City residents consented to be recorded when walking on an unobstructed surface at preferred speed. Gait biomechanics was measured using high speed (100 Hz) motion capture (OptiTrack – Natural Point Inc.), including step length and width, double support, foot contact angle and MFC (swing toe height above the ground). Multivariate Analysis of Variance (MANOVA) was used to confirm ageing effects on MFC and fundamental gait parameters. Pearson’s correlations were performed to identify the relationships between mean MFC and other MFC characteristics (SD and SI), step length, step width, double support time and foot contact angle.

**Results:**

Compared to 50s, lower step length was seen (2.69 cm and 6.15 cm) for 60s and 70s, respectively. No other statistical effects were identified for spatio-temporal parameters between the three groups. The 50s cohort MFC was also significantly higher than 60s and 70s, while step-to-step MFC variability was greater in the 70s than 50s and 60s. Pearson’s correlations demonstrated that more symmetrical gait patterns were associated with greater MFC height, as reflected in greater symmetry in step width (50s), MFC (60s) and foot contact angle (70s). In the 70s increased MFC height correlated with higher MFC variability and reduced foot contact angle.

**Conclusions:**

MFC height reduces from 60 years but more variable MFC appears later, from 70 years. While symmetrical gait was accompanied by increased MFC height, in the 70s group attempts to increase MFC height may have caused more MFC variability and lower foot contact angles, compromising foot-ground clearance. Assessments of swing foot mechanics may be a useful component of community falls prevention.

## Introduction

In demographically ageing societies it is increasingly important to promote healthy ageing to reduce healthcare costs and ensure financially sustainable social security systems. About one in three senior adults fall at least once a year and up to 20 % of cases lead to serious injury or fatality [1]. Previous studies have identified tripping as the leading cause of falls [2, 3] due to the swing foot striking the walking surface, or an object on it, with sufficient force to cause balance loss. Research into ageing effects on walking has, therefore, focussed on identifying foot-ground clearance characteristics that increase tripping risk in older people.

**Fig. 1:**
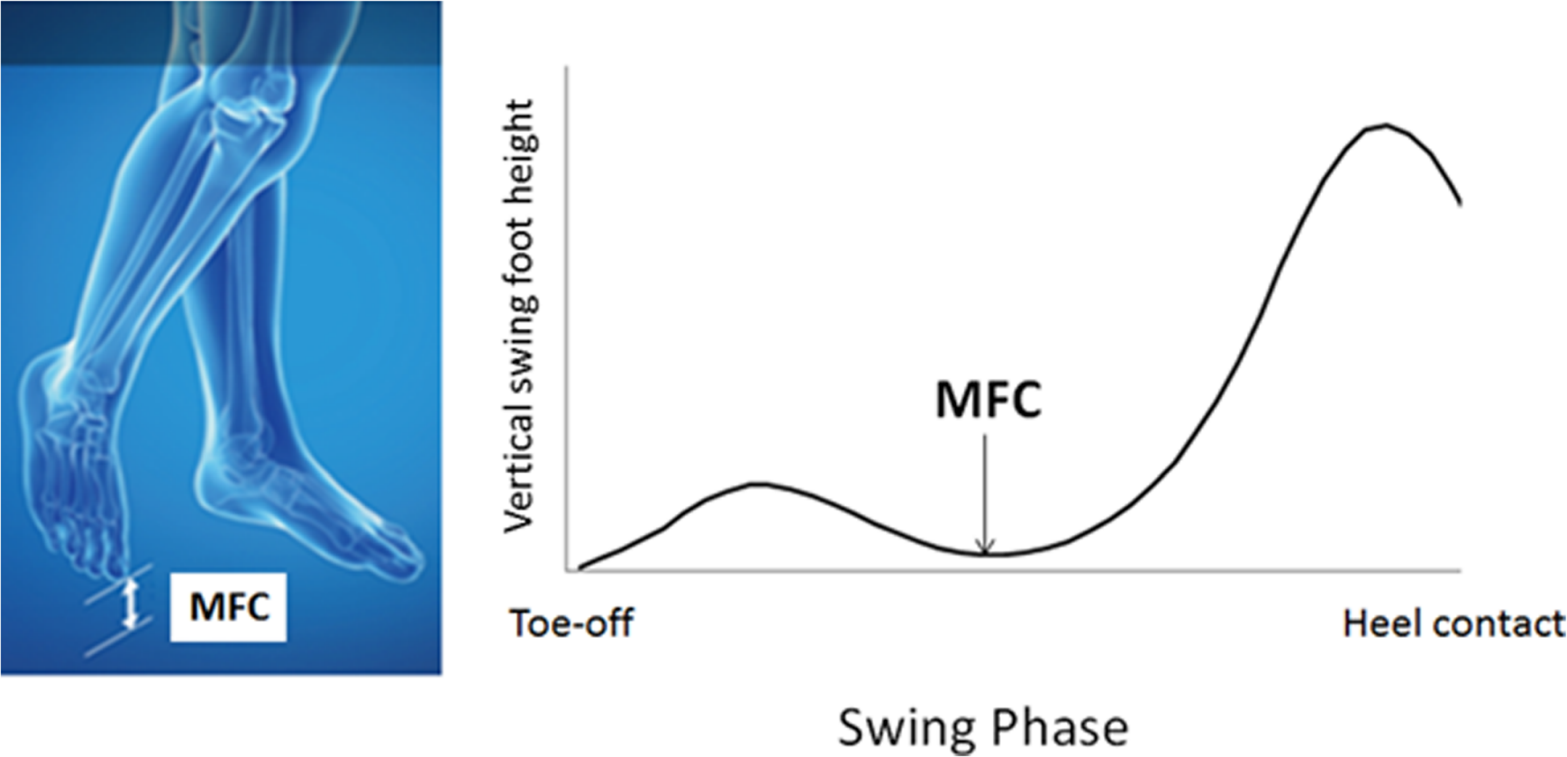
Minimum Foot Clearance (MFC): minimum vertical displacement of the toe at mid-swing. Image taken from Nagano and Begg [16]

Minimum Foot Clearance (MFC) at mid-swing is critical to determining tripping risk associated with undetected obstacles (Fig. 1) [4–15] due to the increased probability of surface contact. At this point, the foot’s forward velocity also approaches maximum, creating a considerable foot-obstacle contact force. Interestingly, in most previous reports, reduced MFC height has *not* been reported to be associated with ageing but greater MFC variability, influencing the MFC distribution, does appear to explain the greater tripping risk as we get older [4]. Analytical approaches to estimating tripping risk have, therefore, modelled the (non-normal) distribution patterns of MFC height and variability [5]. Biomechanically, reduced tripping risk at MFC can be achieved with consistent swing foot clearance across multiple gait cycles (indicated by low variability) combined with low MFC asymmetry [4, 5, 7, 10].

Most previous studies of MFC compared samples of 10 to 30 young adults (18–35 years) with a single group of physically active older people, for example, people over 60 years [6]. For experimental purposes they were, however, undifferentiated with respect to age. The aim of this study was to compare MFC characteristics across three age groups, comprising individuals in their 50s, 60s and 70s. Traditionally, older populations have been defined as above 60 or 65yrs but further investigation of these sub-groups is important in identifying the age-related transition in MFC control from the “pre-old” 50s age group into the 60s and 70s age brackets.

Prolonged life expectancy and lower birth rate in Japan are presenting critical social, economic and public health problems, including mobility and falls. The current study’s focus was older residents of Konosu City, with a population of 120,000 located in Saitama Prefecture Japan, a predominantly rural region of which more than 98 % are Japanese born. More than 30 % are over 65 years, higher than the national average (29 %). While basic gait assessments, such as the 6 minute-walk test [17], are widely used in Japan, there are no reports of ageing effects on gait utilising 3D motion capture. The current research is unique in recruiting a large sample (*n* = 123) of community dwelling citizens outside the laboratory to obtain precise measures of their walking abilities.

Our study was guided by two research questions. First, what are the effects of chronological age on foot-ground clearance, i.e. MFC central tendency, variability (SD) and left-right asymmetry. Symmetrical control of MFC is affected by ageing [7] and based on previous reports (e.g., [4, 7, 8]) it was hypothesised that MFC height would be lower, more variable and also increasingly asymmetrical across the three age groups. The second question was how ageing effects on stride phase variables influence MFC height. This was addressed by correlating spatio-temporal gait cycle variables with MFC height across age groups. Alcock et al. [18] found that slower walking was associated with reduced MFC, possibly due to shorter, wider steps and prolonged double support, all of which are seen with advanced age [19]. Flatter foot-ground contact due to reduced dorsiflexor strength was hypothesised across age groups; a variable that has also been proposed to correlate with lower foot-ground clearance [9, 20].

## Methods

### Participants

Participants were 24 male and 99 female residents of Konosu City (Japan) aged over 50 years, comprising three age groups: 50-59yrs (*n* = 16, age = 56.09 ± 3.44yrs, height 160.2 ± 11.2 cm, body mass 58.5 ± 18.5kg), 60–69 yrs (*n* = 68, age = 65.76 ± 2.67 yrs., height = 156.9 ± 6.01 cm, body mass = 54.45 ± 8.60kg) and 70-79yrs (*n* = 39, age = 74.12 ± 2.57yrs, height 156.3 ± 7.2 cm, body mass 54.6 ± 10.6kg). Sample sizes were larger than most previous studies of ageing effects on MFC [6]. All were classified as healthy, living independently, with no reported locomotor or cognitive impairments. Participant recruitment was initiated by Konosu City Council via advertisements at civic buildings and a mail-out to residents. Prior to participation City Council staff held an information session and all participants gave written informed consent procedures mandated and approved by the institutional research ethics committee of the University of Tsukuba (Tai/30–53).

### Apparatus and procedure

**Fig. 2:**
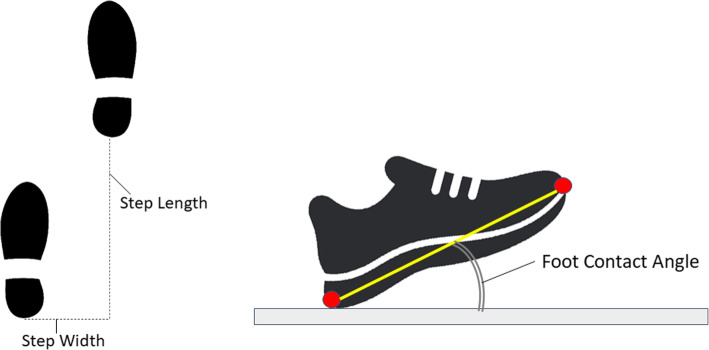
Step Length and Step Width (left) and Foot Contact Angle, the angle formed between the toe-heel axis and the walking surface(right). Red dots represent the toe and heel markers

Using a protocol similar to a pervious study [19] testing was conducted on a 10m walkway in the City Sports Gymnasium at preferred speed, with walkthrough trials repeated until a minimum of 30 complete step cycles had been collected. To model foot motion reflective markers were attached to the toe (the superior most distal surface of the foot) and the heel (the most proximal point) [19]. The reflective marker 3D position-time coordinates were sampled at 100 Hz using a three-dimensional (3D) camera system (Optitrack, Natural Point) and then smoothed at 6 Hz using a low-pass Butterworth digital filter [9]. Toe-off and heel contact were identified by applying conventional gait event detection algorithms to the heel and toe velocity and acceleration functions [7]. MFC was computed as the toe vertical local minimum within a time-sample window mid-swing, using an in-house algorithm implemented in Visual3D (C-Motion, Inc.) script language [5]. Gait variables were computed from the 3D position-time coordinates of the toe and heel markers with step length and width defined, respectively, as anterior-posterior and medio-lateral displacements between the heels at heel contact (Fig. 2). Double support time was the period for which both feet were in contact with the walking surface, i.e. from one heel contact to the contralateral toe-off. Foot contact angle was defined relative to toe and heel positions at heel contact and the walking surface.

As illustrated in Fig. 1, Minimum Foot Clearance (MFC) was the vertical displacement of the toe from the walking surface during mid-swing [4]. All gait data were described using the Mean (central tendency), Standard Deviation (SD) (intra-subject variability) and a Symmetry Index (SI) reflecting left-right differences in the gait variables [7, 21]; computed as follows:

$$ \mathrm{SI}=\left|\left(R-L\right)\left(R+L\right)\times 0.5\right|\times 100\left(\%\right) $$

where R/L indicates right/left foot leading (Fig. 2). The SI was computed for both the mean and SD of each gait variable.

### Design and analysis

In an independent measures design, a One-way Analysis of Variance (ANOVA) was used to determine between-group differences in age, height and body mass. Multivariate ANOVA (MANOVA) was used to confirm ageing effects on MFC and fundamental gait parameters. Significant overall ANOVA effects were followed up using Tukey’s procedure to determine differences between the three age groups. Pearson’s correlations were performed to identify the relationships between mean MFC and other MFC characteristics (SD and SI), step length, step width, double support time and foot contact angle. For all test statistics, i.e., F-ratios, Tukey’s test, and Pearson’s r, significant effects were accepted when *p*-values were less than 0.05.

## Results

One-way ANOVA results confirmed that the three groups were distinguished only by increased age (F_2, 121_ = 278.8, *p* < .01) with no differences in height and body mass.

### Age effects on Gait Parameters

**Fig. 3:**
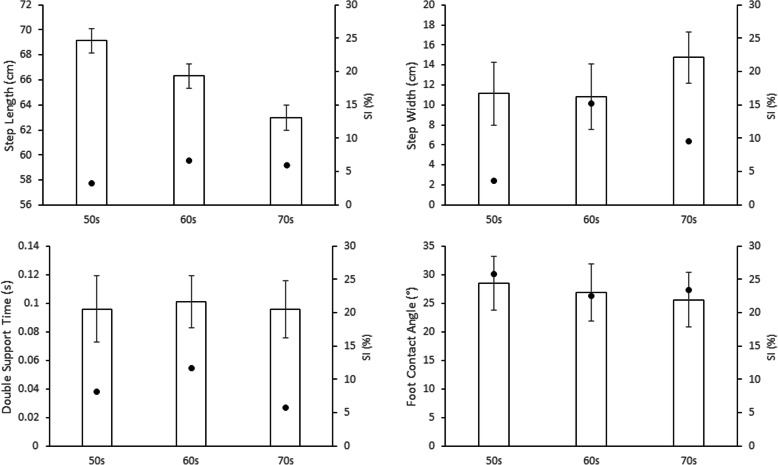
Gait parameter Mean, SD and SI for three age groups. Standard deviation bars indicate intrasubject SD, dots indicate SI shown on the right axis

As expected, age effects were observed for Mean Step Length (F _2, 121_ = 3.309, *p* < .05) due to the 50s group having steps 2.69 cm and 6.15 cm longer than 60s and 70s individuals respectively (Fig. 3).

## Age effects on MFC

**Fig. 4:**
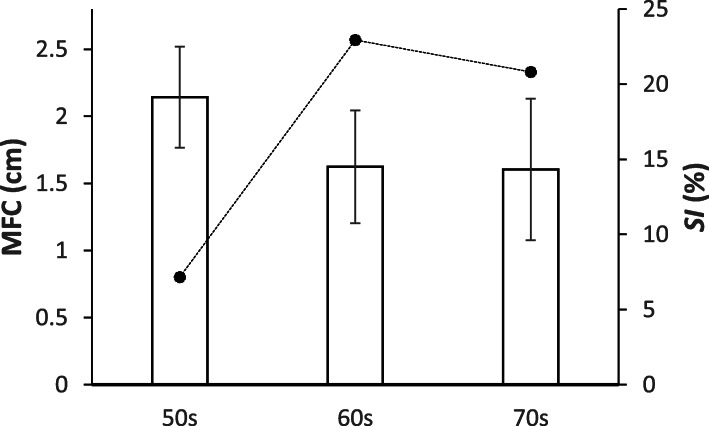
Minimum Foot Clearance (MFC) Mean, SD and SI for three age groups, with standard deviation bars and SI as Fig. 3

MFC characteristics (Mean, SD, SI) of the three age groups (i.e. 50s, 60s, 70s) are displayed in Fig. 4. Ageing effects were observed on Mean MFC (F _2, 121_ = 3.838, *p* < .05) and SD MFC (F _2, 121_ = 4.023, *p* < .05). Tukey’s test revealed differences in Mean MFC between 50s and 60s (*p* < .05) and 50s-70s groups (*p* < .05) due to an approximately 0.5 cm lower MFC in the 60s and 70s participants, as shown in Fig. 4. No Mean MFC difference was found between the 60s-70s groups. In contrast, SD MFC of the 70s age group was 0.149 cm greater than for the 50s (*p* < .05) and 0.106 cm greater than the 60s cohort (*p* < .05) but the 50s-60s comparison did not reveal a difference in MFC variability. SI MFC was lowest in the 50s age range but no statistically reliable age effects on SI MFC were confirmed.

The correlations in Table 1 indicated that across all age groups more symmetrical gait was associated with greater foot-ground clearance, i.e., higher Mean MFC. For the 50s group step width symmetry was associated with higher Mean MFC, in the 60s group MFC symmetry was highly correlated with Mean MFC and in the 70s cohort more symmetrical foot contact angles were significantly associated with higher MFC. Contrary to expectation, in the 70s participants higher SD MFC and greater (more dorsiflexed) Foot Contact Angles were found with *decreased* Mean MFC.

**Table 1:** R values for Pearson’s correlations between gait variables and mean-MFC height (*P < .05, **p < .01). *SD *standard deviation, *SI *Symmetry Index

Age Group	50s			60s			70s		
	**Mean**	**SD**	**SI**	**Mean**	**SD**	**SI**	**Mean**	**SD**	**SI**
MFC		0.323	− 0.007		− 0.032	− 0.320**		.595**	− 0.072
Step Length	0.340	− 0.026	− 0.132	0.212	− 0.073	0.095	− 0.169	0.248	0.018
Step Width	− 0.022	− 0.162	− 0.599*	− 0.105	0.055	0.033	0.065	0.174	− 0.245
Double Support Time	− 0.312	− 0.181	− 0.371	− 0.185	− 0.113	− 0.199	− 0.257	0.021	0.255
Foot Contact Angle	− 0.108	− 0.131	− 0.018	− 0.062	− 0.205	0.201	− 0.477**	− 0.143	− 0.382*

In achieving less MFC variability i.e., lower SD MFC, lower SD Step Length for 50s (*r* = .583, *p* < .05); increased Mean Step Length (*r* = − .240, *p* < .05), reduced SD Step Length (*r* = .250, *p* < .05) and increased Foot Contact Angle (*r* = − .352, *p* < .01) were associated among the 60s; while for the 70s, prolonged Double Support Time (*r* = − .498, *p* < .01) and symmetrical Foot Contact Angle (-0.317, *p* < .05) were identified.

For symmetrical MFC control, symmetrical Step Length showed interlinks for 60s participants (*r* = .370, *p* < .01) and 70s (*r* = .395, *p* < .05) but not 50s (*p* = .105). Symmetrical Double Support Time coincided with symmetrical MFC for 50s (*r* = .601, *p* < .01) and 60s (*r* = .495, *p* < .01) but not for 70s (*p* = .105). Symmetrical control of Step Width was further correlated with SI MFC only for the 60s group (*r* = .345, *p* < .01).

## Discussion

The sampled population was identified as healthy, they were motivated to volunteer for a research project and their fundamental gait parameters were comparable to previous studies with similar age-group samples. Doyo et al. [22], for example, also observed a reduction in step length with age, based on a sample of 2006 community dwelling citizens in Japan. They also reported similar age-related changes in spatio-temporal parameters in the 70s age range, such as reduced step length.

Contrary to previous findings [4, 6, 7] age effects on MFC height *were* found because mean MFC height was significantly lower in the 60s and 70s than the 50s group. Our MFC findings here suggest that MFC height may begin to fall from the 60s, while significantly more variable MFC may appear later, in the 70s age group. Loss of ability to maintain consistent MFC height over multiple gait cycles has been identified as the primary ageing effect on foot trajectory control leading to increased tripping risk [4]. In addition to ageing effects on mean and SD of MFC, the previous study reported that symmetrical control of MFC can be impaired with age [7]. Pearson’s correlations indicated that across all three age groups more symmetrical gait, reflected in a lower SI, was generally associated with elevated MFC height.

Obtained MFC data (central tendency and its variability) were comparable to the previous reports [4–13] but ageing effects were identified unlike these past studies. Most previous research compared young (e.g. 18–35 yrs.) with a single older group (e.g. > 60 yrs.) [6, 10] but our analysis from a considerably larger sample, incorporating three sub-groups, revealed that MFC height may reduce prior to the 60 years age range. In addition to mean and SD descriptions, previous work [7, 19, 20, 23] suggested that reduced leg strength with ageing leads to higher asymmetry, causing loss of symmetrical gait control and increased tripping risk. This is consistent with the current correlations results implying that higher MFC is associated with symmetrical gait in general. In terms of correlations between step length and MFC height, however, in contrast to Alcock et al. [18], we did not find significant correlations but they examined both preferred speed and fast walking, revealing increased MFC due to higher velocity, while the current experiment was conducted only at preferred speed. It may, however, be reasonable to suggest that decreased step length associated with ageing-related declines in walking speed [11, 24] is causally related to reduced MFC height with ageing.

While lower MFC height was identified from the 60s, correlation analysis revealed that ankle control may decline later, from the 70s. This age group showed a positive correlation between mean MFC and SD of MFC, such that the positive effects of elevated MFC were counteracted by increased MFC variability [13]. With ageing, the loss of finely coordinated ankle movement may require a greater contribution from the knee and hip, but these joints are less adapted to precise swing foot control [14, 25]. Increased Foot Contact Angle was also linked to reduced MFC height only in the 70s group, also demonstrating impaired ankle action. Heel contact was associated with dorsiflexion but correlation results suggested that attempts to achieve increased foot contact angle may have caused reduced MFC in the 70s participants.

Reduced MFC height was seen from age 60 years while MFC variability increased from 70 years. While each decade showed different strategies to control MFC, in general, less variable and more symmetrical gait optimises MFC control. Exercise interventions may help in maintaining foot elevation and reducing tripping risk and in addition to maintaining ankle dorsiflexion, particular at mid-swing close to MFC [25], exercises for older people should emphasise symmetrical walking. Treadmill-based gait training with real-time biofeedback, for example, can increase MFC height while reducing variability [12, 26] and gait-feedback provided by “smart footwear” systems may also reduce tripping risk by alerting the wearer to asymmetrical gait control [27, 28].

Precise gait measures obtained using motion capture will more reliably identify age-associated changes to mobility than more commonly used assessments, such as the 6minute-walk test [17]. Large-sample community-based gait screening could also be practically undertaken using a force-sensitive commercial gait assessment system (e.g. GaitRite mat) that does not require specialised skills. For a comprehensive understanding of ground clearance including obstacle negotiation tests, however, 3D analysis is required. Our study used motion capture apparatus, but larger samples could be tested more efficiently using markerless motion capture suits or footwear-mounted wearable sensors. As far as we know, there have been no previous attempts to use 3D motion capture to examine mobility within an everyday community.

Limitations to the current study should be noted. Compared to studies cited in a comprehensive review of the MFC literature [6], the current research incorporated large samples. Further investigation of the same age-defined cohorts is, however, necessary to confirm the present findings of previously unreported ageing effects on MFC height. It is also important to acknowledge that falls risk may be greater in older people who are unwilling to volunteer for a community-based research program. In addition, male participants were underrepresented, possibly due to their lower overall participation in social activities [28, 29]. Further research could be undertaken in communities with different population characteristics to show whether our findings are region-specific. In future work, falls history should also be recorded to investigate whether MFC control is affected by a history of falls. This study was conducted as part of Konosu City’s health promotion initiative and advances in remote gait monitoring, i.e. gait measurement outside the laboratory, will encourage future falls prevention and physical activity initiatives. This early-stage gait assessment scheme should, therefore, be viewed as a community model with the potential to be adopted by other cities to maintain the mobility and safety of their valuable and deserving senior citizens.

## Data Availability

The datasets generated and analyses performed during the current study are not publicly available due to the consent requirement of participants, but anonymous descriptive data are available from the corresponding author on reasonable request.

## Abbreviations

MFC: Minimum Foot Clearance
SD: Standard deviation
MANOVA: Multivariate analysis of variance
